# Subclinical thyroid dysfunctions and the risk of dementia in the oldest old: the Monzino 80‐plus population‐based study

**DOI:** 10.1002/alz.088785

**Published:** 2025-01-09

**Authors:** Ugo Lucca, Mauro Tettamanti, Gianluigi Forloni, Alessia A. Galbussera

**Affiliations:** ^1^ Istituto di Ricerche Farmacologiche Mario Negri IRCCS, Milano Italy

## Abstract

**Background:**

Overt hyperthyroidism and hypothyroidism are regarded as possible causes of reversible dementia. Evidence on the risk of dementia associated with subclinical thyroid dysfunctions is limited and barely existent in the very old, while findings are conflicting. Objective: To cross‐sectionally and longitudinally investigate the association of subclinical‐hyperthyroidism and subclinical‐hypothyroidism with dementia in a prospective population‐based study of 80‐years or older residents in Varese province, Italy (Monzino 80‐plus Study).

**Method:**

TSH, FT4, and FT3 concentrations were determined by immunofluorescence ELFA. According to laboratory reference values of concentration, euthyroidism was defined as a THS between 0.25 and 5.00 mUI/mL, subclinical‐hypothyroidism as a TSH >5.00 mUI/mL and an FT4 within reference range, subclinical‐hyperthyroidism as a TSH <0.25 mUI/mL and an FT4 within reference range. Diagnosis of dementia fulfilled DSM‐IV criteria. Covariates entered in the adjusted logistic regression and Cox proportional hazard models: age, sex, education, alcohol consumption, current smoking, diabetes, hypertension, heart failure, myocardial infarction, COPD, ictus, TIA, parkinsonism.

**Result:**

Consent to blood sampling was obtained from 815 participants, 804 of whom had TSH, FT4 and FT3 measurements available (mean age 90.6 years [SD: 5.3]; 598 women [74.4%]; mean education 5.0 years [SD: 2.4]; 312 with dementia [38.8%]; 710 euthyroid individuals, 33 with subclinical‐hyperthyroidism; 41 with subclinical‐hypothyroidism). At the initial evaluation, neither subclinical‐hyperthyroidism (OR: 1.47, 95%CI: 0.72‐2.97; p = 0.28) nor subclinical‐hypothyroidism (OR: 0.65, 95%CI: 0.31‐1.26; p = 0.21) were significantly associated with dementia. The association remained non‐significant when adjusted for confounders (subclinical‐hyperthyroidism, fully adjusted OR: 1.31, 95%CI: 0.56‐3.02; p = 0.53; subclinical‐hypothyroidism, fully adjusted OR: 0.53, 95%CI: 0.22‐1.18; p = 0.13). At blood sample 418/432 participants with at least one follow‐up available were without dementia. In the following 6 years (median: 2.8 years), euthyroid individuals (N = 403) had similar occurrence of dementia (40.7%) as the 15 with subclinical‐hyperthyroidism (40.0%; fully adjusted HR: 1.08, 95% CI: 0.46‐2.53, p = 0.85) and the 29 with subclinical‐hypothyroidism (41.4%; fully adjusted HR: 0.98, 95% CI: 0.53‐1.84, p = 0.96).

**Conclusion:**

In the present population‐based study, neither subclinical‐hyperthyroidism nor subclinical‐hypothyroidism were significantly associated with the risk of dementia in the very old. Although congruous with previous prevalence estimates, the low frequency of subclinical cases without dementia limited the longitudinal analyses.

